# Investigation of trial registration as part of a research integrity assessment of randomised controlled trials in COVID-19 evidence syntheses: a meta-epidemiological study

**DOI:** 10.1136/bmjopen-2024-092243

**Published:** 2025-05-11

**Authors:** Tamara Pscheidl, Florencia Weber, Emma Sydenham, Patrick Meybohm, Stephanie Weibel

**Affiliations:** 1 Department of Anaesthesiology, Intensive Care, Emergency and Pain Medicine, University Hospital Würzburg, Würzburg, Germany; 2 Cochrane Central Editorial Service, Cochrane, London, UK

**Keywords:** Methods, Systematic Review, STATISTICS & RESEARCH METHODS

## Abstract

**Abstract:**

**Objectives:**

Prospective registration of randomised controlled trials (RCTs) is an international standard of good clinical practice but is often neglected by evidence synthesis producers. This study aims to assess prospective registration of RCTs included in evidence syntheses as part of a research integrity assessment and examine its impact on the study pool.

**Design:**

Meta-epidemiological study.

**Data sources:**

COVID-19 Cochrane reviews (CRs) and non-Cochrane systematic reviews (SRs) in MEDLINE via PubMed up to 9 June 2022.

**Eligibility criteria:**

RCTs from CRs and SRs evaluating 13 investigational medicinal products for SARS-CoV-2 and COVID-19.

**Data extraction and synthesis:**

We assessed prospective trial registration in RCTs according to domain 2 of the research integrity assessment tool. Prospective registration is defined as registration before participant enrolment. We extracted the trial registration number, registration date, study start date and inconsistencies in dates between study report and registration. RCTs were categorised as ‘no concern’, ‘awaiting classification’ and ‘exclude’. We also analysed the relationship between study settings, publishing journals and prospective registration.

**Results:**

We included 188 RCTs. In the primary study report, 91% reported a trial registration number. In 84 RCTs, either not or retrospectively registered or with missing or inconsistent dates, we searched and/or contacted study authors for prospective registrations, resolving 17 RCTs. Ultimately, 58% of RCTs were prospectively registered and considered ‘no concern’, 15% were ‘awaiting classification’ due to inconsistent or missing information and 27% were either not registered or retrospectively registered and categorised as ‘exclude’. Prospective registration was higher in larger or international multicentre RCTs and in RCTs conducted in Europe.

**Conclusions:**

If prospective trial registration is required for inclusion in evidence syntheses, only 6 out of 10 COVID-19 RCTs would be eligible. Restricting eligibility to prospectively registered RCTs would include most large and international multicentre RCTs but exclude many smaller and non-European RCTs.

**Protocol registration:**

The protocol is available on OSF (https://osf.io/3bzeg).

**STRENGTHS AND LIMITATIONS OF THIS STUDY**

- This study comprehensively evaluates the registration status of 188 COVID-19 randomised controlled trials (RCTs), offering a thorough analysis of prospective trial registration practices using the research integrity assessment tool.
- The study incorporates active searches and author inquiries to resolve missing or inconsistent registration details, enhancing the accuracy of the classification process.
- Differences in national and international definitions of prospective registration present challenges in classifying RCTs consistently, impacting the study’s ability to provide a unified assessment.
- The study’s reliance on the submission dates published by ClinicalTrials.gov highlights the limitations of registry transparency as not all registries provide this crucial information, potentially leading to classification errors in prospective registration status.
- This study focuses exclusively on trial registration practices, without considering other factors of research integrity, such as trial conduct, which could also influence the outcomes of evidence synthesis.

## Background

 The basis for reliable results in evidence syntheses is the knowledge of the trustworthiness of the underlying research evidence base. Research that follows the principles of research integrity (RI) ensures trustworthiness. To date, producers of evidence syntheses have not routinely assessed the RI of the studies included in their evidence syntheses. Critical appraisal tools, like the Cochrane Risk of Bias tool 2 (RoB 2), and approaches such as the Grading of Recommendations, Assessment, Development and Evaluation, evaluate the internal and external validity of study results.^1 2^ However, they do not necessarily address aspects of RI. Thus far, there is an ongoing debate on how to appraise RI, and several projects are ongoing to develop trustworthiness screening and RI assessment tools for producers of evidence syntheses.^3^,^5^

Most researchers associate RI with the use of honest and verifiable methods in proposing, performing and evaluating research, but RI also comprises adhering to national, international and commonly accepted guidelines, regulations and norms or standards.^6^ Prospective trial registration is one important international standard for randomised controlled trials (RCTs), which should be discussed for its value in the RI assessment of trials included in evidence synthesis. In 2004 and 2005, the International Committee of Medical Journal Editors (ICMJE) and WHO required prospective trial registration which is defined as registration before enrolment of the first participant.^7 8^ The Declaration of Helsinki has stated that prospective trial registration is required since 2008.^9^ According to the Consolidated Standards of Reporting Trials (CONSORT 2010) statement, information on trial registration should be included when reporting an RCT, that is, item 23.^10^ The key goals of prospective registration are to prevent selective reporting of outcomes and establish a publicly accessible and searchable database for patients and the public, researchers, funders and ethics committees, containing a minimum set of structured information about all ongoing and completed trials.^11 12^ Given the relevance and benefit of prospective trial registration for the public, it is unclear why producers of evidence syntheses have thus far largely ignored when RCTs are not prospectively registered. There is no guidance on how prospective trial registration of RCTs should be assessed and handled within evidence syntheses, and it remains unclear what impact the exclusion of non-registered or retrospectively registered RCTs may have on conclusions of evidence syntheses.

This article is part of a meta-epidemiological study which applies a novel and non-validated research integrity assessment (RIA) tool,^13^ designed for RCTs included in evidence synthesis, to a pool of RCTs included in COVID-19 systematic reviews (SRs). The original RIA tool is available elsewhere.^13^ In the present study, we focus on the assessment of the second domain of the RIA tool, that is, prospective trial registration of RCTs. We present reporting of trial registration in the study reports of COVID-19 RCTs, provide guidance for producers of evidence synthesis on how to assess trial registration in RCTs and discuss the feasibility of the tool for its use in evidence synthesis.

## Methods

The protocol for the meta-epidemiological study has been published, including the search for RCTs and the assessment of prospective trial registration (https://osf.io/3bzeg). We extracted and analysed additional study data which was not prospectively planned, but designed post hoc to describe the study pool in detail. Additional analyses are indicated as such.

### Selection of RCTs for assessment with the RIA tool

We searched for Cochrane reviews (CRs) and non-Cochrane SRs with or without meta-analysis evaluating 13 interventions for the prevention or treatment of SARS-CoV-2 infection and COVID-19 in humans, irrespective of SARS-CoV-2 diagnosis, disease severity or treatment setting. Pairwise and network meta-analyses were eligible. We included full-text, peer-reviewed journal publications of SRs. Preprints of SRs, scoping reviews and narrative reviews were not eligible. We restricted the inclusion to publications in English due to limited resources. Further details on the inclusion criteria of CRs and SRs in terms of population, interventions and comparators are described in the protocol (https://osf.io/3bzeg).

Two reviewers independently searched for all eligible CRs and SRs with regard to study design, population and relevant interventions in PubMed to 9 June 2022. The search strategy is provided in online supplemental file 1. One reviewer selected the CR (or its update) and the SR (or its update) to each of the relevant interventions, with the largest RCT pool based on the most recent search date or the broadest inclusion criteria. The study pool of RCTs which underwent further testing for RIA consisted of the primary studies included in the eligible SRs. RCTs published as journal publications, preprints or unpublished with results posted in trial registries were eligible. We did not combine different reports of the same study (such as journal publications, preprints and trial registration records) identified in the various SRs. Instead, each report was assessed separately as it was included in the original SR.

In the present study, we excluded retracted RCTs (ie, first domain of the RIA) and studies which were incorrectly included in the selected SRs as RCTs, although the studies clearly stated that a non-randomised study design was used. The remaining RCTs were assessed in this study. We documented the screening and selection process of SRs and RCTs in a Preferred Reporting Items for Systematic Reviews and Meta-Analyses (PRISMA) flow diagram, including reasons for exclusion at the full-text screening stage.

### Data extraction of study characteristics

One reviewer (ie, the third reviewer in the meta-epidemiological RIA study, SW) extracted details on trial registration for all RCTs included in this study from the primary study reports, supplemental materials, study protocols and trial registration records up to April 2023. Where available, original data extractions and assessments made by two independent reviewers on prospective trial registration in the RIA study were used and checked by the third reviewer (SW). If double extracted data were not available (ie, for RCTs which previously did not pass domain 1 of the RIA) or if discrepant extractions between pairs of reviewers occurred, a third reviewer (SW) extracted missing data or solved conflicts for this study.

Originally, the second domain of the RIA on trial registration included three items for the assessment of RCTs,^13^ that is, (1) reporting of trial registration with registration number, (2) prospective registration based on the registration date reported in the registration record (eg, date information posted on the registry and date information submitted to the registry) and study start dates reported in the primary study report and in the registration record, and (3) inconsistencies in study dates reported in the primary study report and in the trial registration records. We also extracted the following information of all RCTs, that is, number of identified trial registrations per RCT, study completion date (ie, the longest reported in any study report), sample size, setting (single-centre vs national multicentre vs international multicentre), location (ie, country) where the RCT was conducted and the name of the journal, preprint server or registry where study results were published.

### Assessment of trial registration in RCTs

#### Reporting of trial registration

We investigated whether the RCTs included information on trial registration in the primary study report. To identify trial registration record(s)/number(s), we searched the primary study report (ie, preprint or journal publication) and the study protocol. In cases where we were not able to identify a trial registration number, we actively searched for registration records in national registries, according to the countries where the studies were conducted, and in international registries (eg, International Standard Randomised Controlled Trial Number (ISRCTN), ClinicalTrials.gov). If we could not identify any trial registration, we contacted the study authors. All RCTs for which we were able to ascertain a registration number were categorised as ‘registered RCTs’.

#### Prospective trial registration

We adopted the WHO definition of prospective trial registration, defined as registration before or on the same date of the first participant’s enrolment (eg, study start). Registration after the study start date was deemed to be retrospective registration. We used either the date when the registration was submitted to the registry or when the registration was posted on the registry as the date of trial registration. The submission date of the registration details was only reported on ClinicalTrials.gov. The study start date was extracted from the primary study report and the registration record. In case of missing study start date, we contacted the study authors. In cases of retrospective trial registrations, we actively searched for additional registration records in national registries according to the countries where the study was conducted, and in international registries (eg, ISRCTN, ClinicalTrials.gov), and assessed study start and registration date.

#### Inconsistency in details of study dates

We investigated the consistency of study dates reported in the primary study report and trial registration records. In case of inconsistencies which have an impact on the classification of a prospectively or retrospectively registered study, we contacted the study authors.

### RIA judgement of RCTs considering trial registration

Prospectively registered RCTs without inconsistencies in study reports and registration records were rated as ‘no concern’ (ie, considered eligible for evidence synthesis). Retrospectively registered or non-registered RCTs were rated as ‘exclude’ (ie, considered not eligible for evidence synthesis). If there were any inconsistencies, insufficient information or serious concerns, RCTs were classified as ’awaiting classification’ (ie, considered ineligible for evidence synthesis until clarification).

Authors of the RCTs were contacted if trial registration was not reported in the primary study report, information on study start dates was missing or in case of inconsistencies between study report and registration record. Authors of unpublished RCTs (ie, only trial registration records available) were not contacted since those studies cannot be adequately assessed with current RIA items comparing journal publications or preprints with trial registration records. Authors had 14 days to respond. If a study author provided complete information and confirmed prospective registration, the RCT was upgraded to ‘no concern’. Study authors who did not provide any feedback were reminded via email and given an additional 7 days to reply. The categorisation of the RCTs remained ‘awaiting classification’ if incomplete or no response was received.

### Assessment of the journal policies, indexing for MEDLINE and potentially predatory behaviour

In this study, we also extracted and assessed details of the journal which published the RCTs of interest. This assessment was not included in the original RIA. Additionally extracted and assessed items did not change the RI assessment for the trial registration domain in this study. For all journals that published an eligible RCT for this study, we ascertained if the ICMJE guideline concerning prospective trial registration^12^ was a prerequisite for publication in this journal. We conducted a search from 28 August to 30 August 2023 to check whether the journal was listed on the ICMJE website. The listed date on the website was considered the start date when the ICMJE guidelines were included in the journal’s editorial policies. If the start date or the journal was not listed on the ICMJE website, we gathered the information either by checking the journal’s homepage or by contacting the journal’s editorial team via email. If the information was unavailable or we did not get an email response, we conjectured that these journals do not comply with ICMJE recommendations for prospective trial registration. Next, we compared the date when ICMJE criteria were included in the journals' editorial policies with the publication date of the corresponding RCTs to assess whether following ICMJE guidelines has an impact on the frequency of published prospective registered RCTs. If the information obtained from the homepage or via email contact with regards to prospective trial registration was uncertain, journals were classified ‘unclear’; otherwise, ‘yes’ or ‘no’. If the journal’s policies state that the ICMJE guideline concerning prospective trial registration is only recommended but not binding, the journal policy was classified as ‘not mandatory’. The relevant information from all email responses is provided elsewhere.^14^

Indexing for MEDLINE in the National Library of Medicine (NLM) Catalog,^15^ achieving a high level in the Norwegian Register for Scientific Journals, Series and Publishers (Norwegian Register),^16^ as well as not appearing on Beall’s list^17^ are considered as quality criteria for scientific journals or publishers and were subsequently analysed for all journals publishing eligible RCTs of this study. We conducted a search in the NLM Catalog from 1 September to 8 September 2023. We checked an indexing for MEDLINE by following the link for the journal’s entry in the NLM Catalog, available in the publication’s record in PubMed, or we directly searched for the journal’s name in the NLM Catalog.^15^ Journals that are ‘currently not indexed for MEDLINE’ do not meet all criteria for indexing or are not entitled as a biomedical journal. All indexed journals were assessed by MEDLINE’s Literature Selection Technical Review Committee. Beall’s blacklists for potential standalone predatory journals and publishers were checked by searching the journals' or publishers' names on it from 7 August to 8 September 2023.^17^ Categories ‘yes’ and ‘no’ also apply for the journals listed or not listed on Beall’s list, respectively. Journals and publishers originally listed on Jeffrey Beall’s predatory list, lastly updated 2017, but removed by the present anonymous administrator, retrieved the annotation ‘yes (original Beall’s list 2017)’. We further checked the quality of journals and publishers in the Norwegian Register.^16^ This register has established two ranking lists: one for journals, including standalone journals and journals released by publishers, and one list for publishers only. On 5 October 2023, we checked the journals' levels for the year 2023 by searching the journals' names or International Standard Serial Numbers. Assessment and ranking of journals and publishers have been made by a committee comprised of several experts and can lie between level X, 0, 1 and 2. Journals ranked level 1 or 2 are approved scientific journals from the Norwegian Register. Level 2 comprises journals that fulfil all predefined criteria, and level 1 includes all those which comply with the minimum scientific requirements (eg, external peer review, scientific editorial board and minimum national authorship).^16 18^ A level 0 journal does not satisfy the minimum requirements, hence is considered to be not approved by the Norwegian Register. If a journal was put on the level X list, the committee is in doubt about the scientific quality and uncertain about approval or rejection since researchers reported predatory experiences about them.^16 19^

### Statistical analysis and presentation of data

This study has been designed to facilitate a descriptive data analysis. We did not perform any statistical hypothesis testing as this part of the study was not prospectively planned but designed post hoc to disseminate relevant findings. We compared the categories of RCTs assessed as ‘no concern’ to ‘awaiting classification’ and ‘exclude’, regarding registration details (ie, time from registration or submission to study start), study duration, sample size, setting, location and details on the publishing journal (see above). Descriptive statistics and frequency tables were used to present categorical variables (eg, setting, location, sample size of <100, ≥100 to 200, and ≥200 participants, and journal details). Median and IQRs were calculated for continuous variables (eg, time from registration to study start, time from submission to study start, study duration and sample size).

Due to the large number of studies, we only referenced individual studies in the following results section if less than 10 studies are referred to. Data and digital object identifiers for all individual studies are available online.^14^

### Patient and public involvement

Patients and/or the public were not involved in the design, conduct, reporting or dissemination plans of our research.

## Results

A total of 206 RCTs included in 23 evidence syntheses (ie, 13 CRs and 10 SRs, referenced in online supplemental file 2) investigating interventions of interest for treatment or prevention of SARS-CoV-2 infection were identified by our search. A PRISMA flow diagram is shown in online supplemental file 3. We included 188 RCTs in this study and excluded 8 retracted RCTs and 10 studies which turned out to be non-randomised studies. Of 188 RCTs, 149 were published in journals, 33 were published on a preprint server and the remaining 6 RCTs were unpublished with results only posted on a trial registration database. References and all baseline details of included RCTs reported in the following (ie, trial registration details, sample size, setting, country and journal information) are available elsewhere.^14^

Of 188 RCTs, 165 published RCTs have reported at least one trial registration number in the primary study report (ie, journal publication or preprint), 6 were trial registrations with results not published as article and the remaining 17 RCTs did not report any trial registration number in the primary study report (table 1).

**Table 1:** Reporting and identification of trial registration details in RCTs (n=188)

Registration details	RCTs, n (%)
Reporting of registration number (n=188)	
Reported in primary study report*	165 (88%)
Not reported in primary study report*	17 (9%)
Registrations identified by active search/author request	5
No registration identified	12
Not published, only registration record available as primary study report*	6 (3%)
Number of registrations per RCT (n = 176†)	
One registration record	116 (66%)
Two registration records	49 (28%)
Only one registration number reported in the publication (second identified by active search)	36
All reported in the publication	13
≥ 3 registration records	11 (6%)
Only up to two registration numbers reported in the publication (third identified by active search)	9
All reported in the publication	2
Registry (location) (n = 249‡)	
ClinicalTrials.gov (USA, international)	142
EUCTR (European Union)	56
ISRCTN (UK, international)	15
IRCT (Iran)	12
ChiCTR (China)	9
CTRI (India)	6
ReBEC (Brazil)	5
REec (Spain)	2
INA (Indonesia)	1
SCTR (Saudi Arabia)	1

* Primary study report=publication/preprint or registration record, if RCT unpublished.

† Registrations identified via publication, active search or author request.

‡ The number of registrations exceeds the total number of RCTs due to multiple registrations per RCT. We identified a total number of 249 on 176 RCTs.

ChiCTR, Chinese Clinical Study Register; CTRI, Clinical Trials Registry India; EUCTR, EU Clinical Trials Register; INA, Indonesia Clinical Research Registry; IRCT, Iranian Registry of Clinical Trials; ISRCTN, International Standard Randomised Controlled Trial Number; RCTs, randomised controlled trials; ReBec, Brazilian Registry of Clinical Trials; REec, Spanish Clinical Study Registry [Registro Español de Estudios Clínicos]; SCTR, Saudi Clinical Study Registry.

Of the 17 RCTs which did not report any trial registration number in the publication, we actively searched in national and international trial registries and contacted the study authors to identify trial registrations. Active searching for trial registrations helped classify four RCTs, one as prospective (Sekhavati-2020) and three as retrospective registrations (Chachar-2020, Chowdhury-2021, Purwati-2021). Two of the registrations were identified in national registers and the other two in ClinicalTrials.gov. Author requests for the remaining 13 RCTs helped to classify one RCT as retrospective registration (Mareev-2021), and one study author confirmed that the RCT has not been registered (Podder-2020). 11 of 13 (77%) study authors did not respond to our request, were not available or did not provide sufficient details on trial registration.

Of the 165 RCTs reporting at least one registration number in the publication, initially, 98 RCTs were prospectively registered, 36 were retrospectively registered and 31 had inconsistencies or missing information. Active searches for additional trial registrations in the 36 retrospectively registered RCTs helped classify three RCTs with additional registrations in EU Clinical Trials Register (EUCTR) as prospective registrations (Gupta-2021a, Gupta-2021b, Hermine-2021). Author requests for the 31 studies with missing or inconsistent information helped to classify seven studies as prospective registrations (AlQahtani-2021, Baldeón-2022, Bégin-2021, Kirti-2021, Salama-2021, Sancho-López-2021, Somersan-Karakaya-2022) and two studies as retrospective registrations (Corral-Gudino-2021, Gonzalez-2021). 21 of 31 (71%) study authors did not respond to requests or were not available.

Altogether we investigated 84 RCTs, with an active search for additional trial registrations in 53 RCTs and author requests in 44 RCTs with a response rate of 25%. Finally, 176 RCTs were deemed as registered RCTs, whereas the 12 RCTs without any identified registration were referred to as ‘not registered’.

The majority of the 176 registered RCTs were registered in at least 1 of 10 national or international clinical trials registries, most frequently in ClinicalTrials.gov, followed by the EUCTR and the UK’s trial register ISRCTN (table 1). 116 RCTs were registered once, while 49 RCTs were registered twice and 11 RCTs three times or more (table 1). The second and third registration records were mostly not reported in the publications, but were identified via records in ClinicalTrials.gov or ISRCTN, or the study protocol (table 1).

After completion of our investigations, we assessed 109 RCTs as prospectively registered based on the dates provided in trial registration records and publications, and classified these RCTs as ‘no concern’. In 25 of 109 RCTs, prospective registration could only be identified based on the date of submission to the registry rather than the registration date of the trial registration record. Two RCTs had a retroactively prospective registration according to history data with a change in study start date from retrospective to prospective during the course of the study (Alemany-2022, Ramakrishnan-2021). RCTs considered ‘exclude’ comprise 12 RCTs which were not registered and 39 retrospectively registered RCTs. Among the 39 retrospectively registered RCTs, 16 were registered within 30 days after study start, 12 were registered after 30 days (but before study completion) and 11 were registered after study completion. Of 28 RCTs held in ‘awaiting classification’, 13 had inconsistent information on study start dates between publication and registration record, 8 had missing information on study start dates (Derde-2021, Entrenas Castillo-2020, Farahani-2020, Jamaati-2021, Li-2021, Portal-Celhay-2021, Rastogi-2020, Stone-2020), 6 were unpublished but registered trials (CJWT629A12301, NCT04335552, NCT04385199, NCT04392141, NCT04407507, NCT04421404) and 1 had an inaccessible registration record (Sabicio-2021).

The median time between registration and study start (time point=0) varied: −3 days (IQR −10 to 0) for prospectively registered RCTs, 2 days (IQR −3 to 12) for unclear registrations and 41 days (IQR 15 to 101) for retrospectively registered RCTs (table 2). Prospectively registered RCTs had more participants and longer study durations than non-registered or retrospectively registered RCTs or RCTs held in ‘awaiting classification’ (table 2). In large RCTs (≥ 200 participants), 83% were prospectively registered compared with 25% of small RCTs (<100 participants) (table 2). Among 'no concern' RCTs, 14% had fewer than 100 participants; in 'awaiting classification' and 'exclude' groups, 18% and 20% had 200 or more participants (table 2). 97% of international multicentre RCTs and 64% of national multicentre RCTs were prospectively registered, while only 30% of single-centre RCTs were (table 2). In 'no concern' RCTs, 18% were single-centre; in 'awaiting classification' and 'exclude' groups, 4% and 0% were international multicentre. In Europe, 83% of national multicentre and single-centre RCTs were prospectively registered compared with about 50% in South and North America, and about 30% in Asia and Africa (table 2). Half of 'awaiting classification' and 47% of 'exclude' RCTs were conducted in Asia.

**Table 2:** Characteristics of randomised controlled trials classified as ‘no concern’, ‘awaiting classification’ and ‘exclude’ (n=188)

Study characteristics	No concern (n=109)	Awaiting classification (n=28)	Exclude (n=51)
Time between registration and study start (days)*			
Median (IQR)	−3 (−10 to 0)	2 (−3 to 12)	41 (15 to 101)
No information	0	1	0†
Time between submission and study start (days)*			
Median (IQR)	−8 (−17 to −4)	−3 (−13 to 5)	23 (9 to 88)
No information	22	12	9†
Study duration (days)*			
Median (IQR)	281 (114 to 723)	129 (72 to 254)	114 (76 to 187)
No information	10	1	4
Sample size; randomised participants			
Median (IQR)	400 (131 to 799)	68 (33 to 124)	89 (58 to 155)
<100 participants (n=60)	15	19	26
100 to <200 participants (n=40)	21	4	15
200 or more participants (n=88)	73	5	10
*Setting and location*			
Multicentre, international (n=32)	31	1	0
Multicentre, national (n=90)	58	10	22
Asia	9	5	8
Europe	30	0	6
North America	9	5	4
Africa	1	0	0
South America	9	0	4
Singlecentre (n=66)	20	17	29
Asia	8	9	16
Europe	4	1	0
North America	2	3	1
Africa	2	2	5
South America	4	2	7

* According to dates from the registration record; in case of multiple registrations, we used the trial registration record referenced in the publication. Time was measured between submission/registration and study start. Study start was defined as time point 0. Negative days indicate ’registration/submission before study start’ and positive days indicate ’registration/submission after study start’.

† Only registered studies and according to registration record.

Of 188 RCTs, 149 published in journals were analysed: 90 were prospectively registered ('no concern'), 15 had inconsistent/missing data ('awaiting classification') and 44 were not or retrospectively registered ('exclude') (table 3). In ICMJE-compliant journals, 69% of RCTs were prospectively registered compared with 26% in non-ICMJE journals (table 3). Among 'no concern' RCTs, 91% were published in ICMJE-compliant journals vs 60% of 'awaiting classification' and 61% of 'exclude' RCTs. In MEDLINE-indexed journals, 64% of RCTs were prospectively registered compared with 40% in non-indexed journals (table 3). Among 'no concern' RCTs, 91% were published in MEDLINE-indexed journals compared with 93% of 'awaiting classification' and 75% of 'exclude' RCTs. None of the RCTs was published in a level X Norwegian Register journal. One journal (ie, *Internal and Emergency Medicine*) that published an RCT (Pouladzadeh-2021) assessed as ‘exclude’ was ranked level 0. Of 78 RCTs published in level 2 journals, 87% were prospectively registered compared with 21% of 56 RCTs in level 1 (table 3). Among 'no concern' RCTs, 76% were published in level 2 journals compared with 7% of 'awaiting classification' and 20% of 'exclude'. 14 journals were unlisted or not assessed, publishing 11 'exclude' and 3 'no concern' RCTs. Four journals (ie, *International Journal of Science, Paripex Indian Journal of Research, Nutrients* and *Journal of Clinical Medicine*) on Beall’s list published five RCTs: three either not or retrospectively registered (Chachar-2020, Kishoria-2020, Sánchez-Zuno-2021), one prospectively registered (Song-2021) and one 'awaiting classification' (Sabico-2021) (table 3).

**Table 3:** Journal characteristics publishing RCTs classified as ‘no concern’, ‘awaiting classification’ and ‘exclude’ (n=149)

Journal characteristics	No concern (n=90)	Awaiting classification (n=15)	Exclude (n=44)*
Adherence to ICMJE recommendations			
Published by journal following ICMJE recommendations†	82	9	27
Published at or after the date the journal starts to follow ICMJE recommendations	74	5	22
Published before the date the journal starts to follow ICMJE recommendations	1	0	2
Unknown when the journal starts to follow	7	4	3
Published by journal not (mandatorily) following ICMJE recommendations† or with insufficient information	8	6	17
MEDLINE indexing			
Published by journal indexed for MEDLINE	82	14	33
Published by journal currently not indexed for MEDLINE	8	1	11
Level within the Norwegian Register‡			
Level X	0	0	0
Level 0	0	0	1
Level 1	19	14	23
Level 2	68	1	9
Not listed or currently not assessed	3	0	11
Listed on Beall’s list			
Published by journals/publishers not listed on Beall’s list	89	14	41
Published by predatory journals/publishers according to Beall’s list	1	1	3

* Including 33 retrospectively registered RCTs and 11 non-registered RCTs.

† Information on homepage/via email contact regarding prospective trial registration or journal listed on ICMJE list.

‡ The Norwegian Register’s ranking system includes levels X, 0, 1 and 2. Level 1 and 2 journals are approved, with level 2 meeting all criteria and level 1 meeting the minimum requirements. Level 0 journals do not meet the standards, while level X indicates uncertainty due to concerns about predatory practices.

ICMJE, International Committee of Medical Journal Editors; RCTs, randomised controlled trials.

## Discussion

In our assessment including 188 COVID-19 RCTs, 9 out of 10 reported at least one trial registration number, and 1 in 10 RCTs did not report any registration details. Active searches or author requests in 84 RCTs, which were either not or retrospectively registered or with inconsistent or missing information on study start dates, resolved about 20% of cases, resulting in 11 prospective and 6 retrospective registrations. Ultimately, only 58% of the 188 RCTs were prospectively registered and fully eligible for evidence synthesis according to the RIA tool. The remaining RCTs were deemed not eligible for evidence synthesis due to lack of registration, retrospective registration, missing information or inconsistencies.

Nevertheless, our study showed a substantial increase in prospective trial registration in COVID-19 studies compared with earlier years.^20 21^ Al-Durra *et al*, for example, investigated about 10 000 manuscripts of RCTs published in more than 2000 journals in 2018 and found that 42% complied with prospective trial registration.^20^ In the context of RIA, evidence syntheses examining RCTs published before the COVID-19 pandemic would include even fewer prospectively registered studies, resulting in an even smaller study pool.

Definitions of prospective registration vary nationally and internationally, hampering classification for evidence synthesis producers. Among the 39 retrospectively registered RCTs, 16 were registered within 30 days after the study start, aligning with US and UK regulations.^22 23^ In contrast, we used the international WHO and ICMJE definition of prospective registration which means registration before enrolment of the first participant.^7 8^ In this respect, international harmonisation of clinical trials regulation would be helpful for classification.

Additional challenges in assessing trial registration include inconsistencies in study dates between registration and publication as well as multiple registrations or unclear primary sites in multicentre RCTs. Reporting of trial registration details, including study dates and primary sites, should be improved. ClinicalTrials.gov is the only registry publishing submission dates. In 20% of the RCTs, prospective registration could only be identified based on the submission date. Submission dates are crucial for accurate classification as delays in processing submissions can be expected during crisis times, such as the COVID-19 pandemic. We suggest that all clinical trial registries should publish submission dates of complete registrations.

Two RCTs in our sample changed their study start date at later time points, altering their classification from retrospective to prospective registration. A recent study measured the rate of ‘retroactively prospective’ trials in ClinicalTrials.gov in 2015^24^ and identified 2% of all clinical trials in a sample of 11 908 trials. While these changes to the start date could be mistakes or legitimate edits based on the most up-to-date information, they could also indicate a retrospectively registered trial that has been made to appear as a prospectively registered trial, which represents scientific flaw and would lead to biases unapparent to producers of evidence syntheses.^24^ For RI assessments in evidence synthesis, we need a consensus on handling 'retroactively prospective' RCTs in evidence synthesis.

We contacted the authors of 44 RCTs that either lacked a reported trial registration number or had inconsistent or missing information regarding registration or study dates. However, the response rate was only 25%. Out of the 11 RCTs that did respond, 7 could be classified as prospectively registered. This suggests a risk that a significant number of inconclusive RCTs are prospectively registered but may have been incorrectly excluded in the RIA. We believe that responsiveness in correspondence is a key indicator of trustworthiness, while a lack of response undermines it. Accountability and transparency are crucial for RI. RCTs that fail to transparently report essential trial registration details or refuse to share this information on request raise concerns about their RI.

Producing evidence syntheses can be time-consuming and costly. It is particularly challenging to review poorly reported clinical trials that do not adhere to international standards. How thoroughly should evidence synthesis producers examine these trials? The process becomes even more labour-intensive when it involves contacting authors, searching for additional registrations, clarifying inconsistencies and checking historical data in trial registries. While trial registration is easier to verify compared with other aspects such as ethics approval^25^ or data authenticity—which is nearly impossible to verify without statistical expertise—clear guidance for evidence synthesis producers on the components and extent of the assessment is still needed.

Trinquart *et al* showed higher registration rates for industry-supported and larger RCTs, and Al-Durra *et al* revealed a relation between the prospective registration of clinical trials and the trial registry, region, condition, funding, trial size, interval between registration and paper submission dates, impact factor and ICMJE membership of the publishing journal.^20 26^ In our study, restricting eligibility to prospectively registered RCTs would include 83% of large RCTs and 97% of international multicentre RCTs, but exclude many smaller and non-European studies. We should consider whether this restriction would be useful, particularly for rapid reviews. However, future studies should examine the consequences of such restrictions on the diversity of the evidence base.

In our study, a publication in a journal following the ICMJE recommendation or indexed for MEDLINE is not a reliable indicator for prospective registration as 30–40% of RCTs in such journals are retrospectively registered. Only publication in level 2 journals of the Norwegian Register appears to be associated with prospective registration. Level 2 is the highest level, whereas level 1, where most of the journals publishing the not prospectively registered RCTs were placed, is considered to satisfy the minimum requirement to be counted as scientific (external peer review, scientific editorial board and minimum national authorship).^27^ It should be considered whether the Norwegian Register should be included as an indicator of trustworthiness in an RI assessment.

Our study has several limitations. First, RIA is limited to SRs of more recently conducted RCTs. Second, our study is limited to a RIA of COVID-19 RCTs. Therefore, generalisability to other time periods or other medical fields is limited. Third, lack of statistical testing, considering the absence of prospective planning, is another limitation of this work hindering strong conclusions on any reported association between study characteristics and prospective registration.

We face the challenge of how to handle studies without prospective registration in RI assessments conducted within evidence syntheses. In RIA, all RCTs without prospective registration have been excluded, regardless of other aspects such as ethics or data trustworthiness. We have chosen a hierarchical approach to work more efficiently. This approach was based on the assumption that restricting to prospective RCTs would not result in the loss of large, well-conducted trials. In contrast, TRACT, another trustworthiness checklists, assesses RCTs without prospective registration (and published after 2010) as ‘major concern’ triggering a more thorough investigation, including assessment of original individual participant data.^3^ A third Trustworthiness Screening Tool developed by the Cochrane Pregnancy and Childbirth Group places RCTs without prospective registration (and published after 2010) in the ‘awaiting classification’ category, meaning they do not contribute to evidence synthesis findings.^4^ The key question for the research community is whether the study pool should be restricted to prospectively registered RCTs or whether prospective registration should be viewed as part of a broader, more holistic approach in a RI assessment, encompassing ethics and governance, to prevent the exclusion of relevant RCTs.

Handling non-prospectively registered studies in evidence synthesis can have an educational effect on future RCTs. Since registration is embedded in the CONSORT statement and is an international principle, excluding non-registered studies is justified. However, the definition of retrospective registration is disputed—whether within 30 days to 6 weeks (as in the USA and formerly UK) or only after study completion. The fact is that studies without a prespecified analysis plan (or a protocol at a pinch), which most non-registered or retrospectively registered studies fall into, cannot be reliably assessed for risk of bias with the Cochrane RoB 2 tool,^2^ especially for the domain of selective outcome reporting, giving them theoretically a comparative advantage over prospectively registered studies. Only prospectively registered studies allow for the identification of selective outcome reporting resulting in a ‘high risk of bias’ assessment, meaning that non-registered or retrospectively registered studies can never be rated as ‘high risk of bias’ in this domain.

Today, there is no justification for missing prospective registration. We, as producers of evidence synthesis, must consider this in our RI assessments. A fully reliable study must be prospectively registered. Only when such studies are no longer cited in SRs and guidelines due to non-compliance with international standards, a shift in perspective can be forced, affecting funding and personal reputation. Journals also play a crucial role in the publication of these studies. Strict implementation of ICMJE guidelines could ensure that publication chances are minimised, thereby enforcing prospective registration. Prospective registration can be done with minimal financial and personnel resources from anywhere in the world in national or international registries.

## Conclusion

If prospective trial registration is required for inclusion in evidence syntheses, only 6 of 10 COVID-19 RCTs would be eligible. Reporting of registration details and study dates was insufficient in 15% of RCTs, and 27% of RCTs were not or retrospectively registered. The frequency of prospective registration varies by study setting and country. Restricting eligibility to prospectively registered COVID-19 RCTs would include the vast majority of large RCTs and international multicentre RCTs but exclude many smaller and non-European studies. To our mind, a consensus is needed within the evidence synthesis community on whether a study pool should be restricted to prospectively registered RCTs. Currently, we argue in favour of restricting the study pool to prospectively registered RCTs (in SRs of more recently conducted studies) because it aligns with international standards, is easy for trialists to implement and straightforward for systematic reviewers to assess, is essential for correctly assessing the bias of an RCT and speeds up the evidence synthesis process by excluding many small and poorly reported RCTs.

## Supplementary material

10.1136/bmjopen-2024-092243 online supplemental file 1

10.1136/bmjopen-2024-092243 online supplemental file 2

10.1136/bmjopen-2024-092243 online supplemental file 3

## Data Availability

Datasets are included in this published article, supplementary information files, or available from the OSF repository, DOI: 10.17605/OSF.IO/87UT4.
