# Supplementary material for: Investigation of trial registration as part of a research integrity assessment of randomised controlled trials in COVID-19 evidence syntheses: a meta-epidemiological study
Source: BMJ Open. 2025 May 11;15(5):e092243. doi: 10.1136/bmjopen-2024-092243 (PMC12067809; doi:10.1136/bmjopen-2024-092243)
Supplement: Supplementary file 4 [file bmjopen-2024-092243.reviewer_comments.pdf]

PEER REVIEW HISTORY

BMJ Open publishes all reviews undertaken for accepted manuscripts. Reviewers are asked to complete a checklist review form and are provided with free text boxes to elaborate on their assessment. These free text comments are reproduced below.

ARTICLE DETAILS

Title (Provisional)

Investigation of trial registration as part of a research integrity assessment of randomized controlled trials in COVID-19 evidence syntheses: a meta-epidemiological study

Authors

Pscheidl, Tamara; Weber, Florencia; Sydenham, Emma; Meybohm, Patrick; Weibel, Stephanie

---

VERSION 1 - REVIEW

**Reviewer**	**1**
**Name**	**Siemens, Waldemar**
**Affiliation**	**University Clinic Freiburg**
**Date**	**16-Oct-2024**
**COI**	**None**

Summary

The authors present a study on research integrity of RCTs, especially focusing on the lack of prospective trial registration. The study is well reported. My main concern relates to the conclusion raising the question if not prospectively registered trials should be included in evidence synthesis (see comment 6).

Major comments

#1

Abstract: “We also examined the impact of study settings and publishing journals on prospective registration and discussed the reliability of these assessments”. Is it an impact (causality) or rather an association? Probably the latter.

Methods:

#2

Was a reporting guideline used for this meta-research study? PRISMA?

#3

From my perspective, “meta-research study” should be added in the title as study design; meta-epidemiological study might not fit as well as meta-research study

#4

How was sample chosen? Please add more details. Was the study part of another project? If yes, this should be made more explicit.

#5

Please add a limitations chapter in the discussion.

#6

“To our mind, a consensus is needed within the evidence synthesis community on whether a study pool should be restricted to prospectively registered RCTs. Currently, we argue in favor of this approach because it aligns with international standards, is essential for correctly assessing a RCT, is easy for trialists to implement, and speeds up the evidence synthesis process by excluding many small and poorly reported RCTs.” – #6.1 I would rather be cautious and I don’t think that we should make generic statements like to always exclude not prospectively registered RCTs. Meta-research is needed to show if the excluded studies could change results in meta-analyses. Even if meta-research shows results that these trials might be excluded researchers should still be able to decide that for each individual systematic review as the impact might be very context-specific. I think sensitivity analyses would be a better way to deal with it rather than exclude these trials. #6.2 Please also discuss if a missing protocol or registration is already addressed in RoB 2 or in ROBINS-I (I think yes). Therefore, the risk of bias due to a missing registration/protocol might be already considered and could be reported as such; and also lead to downgrading of the certainty of evidence. Please discuss and revise the conclusion if appropriate.

Minor comments

Methods:

#1

Could you attach the RIA tool to the manuscript so readers would better understand how the tool looks like?

#2

“We documented the screening and selection process of systematic reviews and RCTs in a PRISMA flow diagram including reasons for exclusion at the full-text screening stage.” – add Figure number.

Results:

#3 Table 2: Could add a legend explaining what “minus days” (e.g. -3) mean and what “plus days” (e.g. 2).

#4

Table 2: You could add percentages behind frequencies where appropriate.

#5

Table 3: The title of the table does not fit well to the categories (ICMJE, MEDLINE…). These are not journals.

#6

Table 3: You could add percentages behind frequencies where appropriate.

#7

Table 3: The levels in the Norwegian Register could be explained in the legend.

#8

You could refer to the flow diagram at the beginning of the results chapter.

#9

The discussion would benefit from subchapters.

#10

Conclusions: "If prospective trial registration is required for inclusion in evidence syntheses, only six of ten COVID-19 RCTs would be eligible" – Do you have a comparison from non-Covid studies you could include in the discussion?

#11

Conclusions: "we argue in favor of this approach" – Which approach?

**Reviewer**	**2**
**Name**	**Rahouma, Mohamed**
**Affiliation**	**Weill Cornell Medicine, Cardiothoracic Surgery**
**Date**	**01-Jan-2025**
**COI**	**None**

Pscheidl et al reported their work named "Investigation of trial registration as part of a research integrity assessment of randomized controlled trials in COVID-19 evidence syntheses" and concluded "If prospective trial registration is required for inclusion in evidence syntheses, only six out of ten COVID-19-RCTs would be eligible. Restricting eligibility to prospectively registered RCTs would include the vast majority of large and international multi-centre RCTs but exclude many smaller and non-European RCTs.". I have the following comments:

Main comment:

- Statistical Analysis: The study is described as descriptive, and no statistical hypothesis testing was performed. While this is appropriate for the study's aims, the authors should consider

whether more advanced statistical methods (e.g., regression analysis) could provide additional insights into factors associated with prospective registration.

- Data Availability and Transparency: The authors mention that they contacted study authors for missing or inconsistent registration details. However, the response rate was only 25%. The manuscript should discuss the potential impact of non-response on the study's findings and whether alternative methods could be used to obtain missing data.

Other comments:

- Clarity and Justification of the RIA Tool: The authors use the Research Integrity Assessment (RIA) tool, which is described as novel and non-validated. While the tool appears comprehensive, the manuscript would benefit from a more detailed discussion of its development, validation process, and how it compares to existing tools for assessing research integrity. Additionally, the authors should justify why this tool was chosen over other established methods for assessing trial registration.

- Generalizability of Findings: The study focuses exclusively on COVID-19 RCTs, which may limit the generalizability of the findings to other therapeutic areas. The authors should discuss whether the observed trends in trial registration are likely to be similar in non-COVID-19 trials or if the urgency of the pandemic may have influenced registration practices differently.

- Handling of Retrospective Registrations: The authors classify RCTs as "retrospectively registered" if registration occurs after the study start date. However, they note that some registries (e.g., in the US and UK) allow registration within 30 days of study initiation. The manuscript would benefit from a more nuanced discussion of how different regulatory environments might impact the classification of retrospective registration and whether a uniform standard (e.g., WHO/ICMJE) should be applied globally.

- Impact of Excluding Non-Prospectively Registered RCTs: The authors suggest that excluding non-prospectively registered RCTs would exclude many smaller and non-European studies. While this is an important finding, the manuscript would benefit from a deeper exploration of the potential consequences of such exclusions. For example, how might this impact the diversity of evidence in systematic reviews, and could it lead to biases in the evidence base?

- Journal Policies and Compliance: The authors assess whether journals follow ICMJE recommendations for prospective trial registration but find that 30-40% of RCTs in ICMJE-compliant journals are retrospectively registered. This raises questions about the enforcement of journal policies. The authors should discuss potential reasons for this discrepancy and suggest ways journals could improve compliance with registration requirements.

- Ethical Considerations: The manuscript briefly mentions ethics approval but does not explore the ethical implications of non-prospectively registered trials in depth. Given that prospective registration is an ethical requirement in many jurisdictions, the authors should discuss the ethical dimensions of their findings, particularly in the context of patient safety and transparency.

- Discussion of "Retroactively Prospective" Trials: The authors identify two RCTs that changed their study start dates to appear prospectively registered. This is a concerning finding that warrants further discussion. The authors should explore the potential motivations for such changes and suggest ways to prevent this practice in the future.

- Recommendations for Evidence Synthesis Producers: The authors conclude that a consensus is needed on whether to restrict evidence syntheses to prospectively registered RCTs. While this is a valid point, the manuscript would benefit from more specific recommendations for systematic reviewers. For example, should reviewers always exclude non-prospectively registered trials, or are there circumstances where they might be included with appropriate caveats?

- Limitations: The authors acknowledge several limitations, including the focus on trial registration and the reliance on ClinicalTrials.gov for submission dates. However, they should also discuss the potential impact of these limitations on the study's conclusions and suggest ways future research could address these issues.

**Reviewer**	**3**
**Name**	**Jull, Andrew**
**Affiliation**	**The University of Auckland, School of Nursing**
**Date**	**12-Mar-2025**
**COI**	**None**

Thank you for the opportunity to read this interesting paper. The purpose is to assess whether trial registration is an element that should be considered in assessing the quality of a RCT when including the same in systematic review. I have no major concerns about this paper, but raise a few minor issues that need resolution.

1. Abstract and elsewhere: It is not clear to me whether the conclusions are limited to trials associated with COVID interventions and are more generalisable. I suspect the authors are aiming for the latter, but if so, will need to generalise the conclusions (especially in the abstract), which appear limited to inferences that might be drawn from COVID trials alone. I do think that that if the authors are aiming at more generalisable conclusions as indicated in the conclusions on page 20, then that attempt is precipitate, given the only data then have tested their hypotheses upon are trials involving COVID and other groupings of trials needed to be evaluated before the evidence synthesis community could be asked for a consensus on using prospective trial registration as an inclusion criteria. However, it certainly makes sense to consider prospective trial registration as a marker for sensitivity analyses, but not a for an in/out decision at this point in time.

2. Page 4: GRADE is not a critical appraisal tool, but rather is a means for evaluating the level of certainty associated with an evidence statement guiding the type of language that should be used in the evidence statement. Also why is inter in brackets for international on line 26?

3. Please provide more clarity in the last sentence in paragraph 3 on page 6. I did not understand the point the authors were trying to make.

4. Line 24 page 8 and through out: please spell out in full prospectively instead of "pro- or retrospectively...".

5. Lines 10-12, page 9: I could not understand the sentence - please provide more clarity.

6. Page 13, line 47: The ISRCTN is the UK's trial register and is owned by the the non-profit company ISRCTN and operated by Springer Nature on their behalf.

7. Page 14, line 30: please provide detail of prospective registration for the >100<200 group of trials.

8. Table 2: For setting and location, all locations are multinational continents, except for Australia. Was it your intention to only include Australia or did you mean Australasia (Australia and New Zealand) or Oceania (Australia, NZ, Pacific nations). Further, given there were 0 trials in Australia, why include it at all?

9. Page 16, line 29: revise to read "three either not or retrospectively registered..."

10. Discussion first paragraph and conclusion: please revise page 18 line 7 and page page 20 line 47. It is not the case that every 10th trial did not report registration details or study date insufficient on every 7th trial), which would mean that trials 10, 20, 30, 40, etc (or 7, 14, 21, 28, etc) were affected. Instead you mean one in 10 trials (or one in seven trials) were so affected.

11. Page 18, line 30: Do you mean regulation or registration?

12. Page 19, line 41: is it appropriate to use correlate when no such association has been established with significance testing? Also line 44 - include "is" after placed.

13. Revise page 19, lines 58-60 to read "The key question is not whether prospective registration should be an isolated exclusion criterion, but whether it should be considered..."

14. Page 20, lines 17-21: You raise a reasonable point, but do not consider [a] whether an retrospectively registered trial (or indeed) an unregistered trial with a published protocol has such a theoretical advantage; [b] whether trials published before either registers were available (1999-2000 for ISRCTN and clinical trials.gov) or before registration was announced as mandatory (Sept 2004) and made mandatory for participating journals that made up the ICMJE at the time (Sept 2005); and [c] the Cochrane RoB v2 (RoB2) tool actually asks about whether data was analysed in accord with a pre-specified analysis plan (or a protocol at a pinch), which are rarely available on trials registration pages and thus simply comparing lists of outcomes in a publication and a trials registry is insufficient to gain a pass in RoB2, so most trials will not gain a theoretic advantage according to the RoB2 algorithm (https://drive.google.com/file/d/1Q4Fk3HCuBRwIDWTGZa5oH11OdR4Gbhdo/view) and will be scored "some concerns" on the selected outcome domain leading to an overall assessment of some concerns even if all other domains are low risk. So I believe that the case you make is an overstated risk.

VERSION 1 - AUTHOR RESPONSE

Reviewer: 1

Dr. Waldemar Siemens, University Clinic Freiburg Comments

to the Author:

Summary

The authors present a study on research integrity of RCTs, especially focusing on the lack of prospective trial registration. The study is well reported. My main concern relates to the conclusion raising the question if not prospectively registered trials should be included in evidence synthesis (see comment 6).

Thank you for your critical and helpful comments.

Major comments:

#1 Abstract: "We also examined the impact of study settings and publishing journals on prospective registration and discussed the reliability of these assessments". Is it an impact (causality) or rather an association? Probably the latter.

Response: Thank you for your comment. We changed "impact" into "relationship". It now reads as "We also analyzed the relationship between study settings, publishing journals and prospective registration."

Methods:

#2 Was a reporting guideline used for this meta-research study? PRISMA?

Response: Yes, we used a reporting guideline. We reported on this in our protocol:

"This is a protocol to a meta-epidemiological study following reporting guidelines. (Ref 1) We adopt a systematic review approach. The unit of analysis in meta-epidemiological studies is a study, not a patient. (Ref 1)"

(Ref 1) Murad MH, Wang Z. Guidelines for reporting meta-epidemiological methodology research. Evid Based Med. 2017;22(4):139-142. doi:10.1136/ebmed-2017-110713

#3 From my perspective, "meta-research study" should be added in the title as study design; meta-epidemiological study might not fit as well as meta-research study

Response: Thank you for your comment. We agree that the title should clearly reflect the study design. However, we believe that our study aligns with the meta-epidemiological study design as

defined by Murad et al. (Ref 1). We have already published another paper related to that study using the term "meta-epidemiological study," and we want to avoid confusing readers who are familiar with that work. The previous paper is titled "Investigation of ethics approval as part of a research integrity assessment of randomized controlled trials in COVID-19 evidence syntheses: A meta-epidemiological study" (BMJ Open 2025;15:e092244. doi: 10.1136/bmjopen-2024-092244).

We have revised the title to reflect these considerations: "Investigation of trial registration as part of a research integrity assessment of randomized controlled trials in COVID-19 evidence syntheses: a meta-epidemiological study"

#4 How was sample chosen? Please add more details. Was the study part of another project? If yes, this should be made more explicit.

Response: We apologize for the lack of clarity. The present study is part of a larger meta-epidemiological study which is unpublished. We referenced the protocol (https://osf.io/3bzeg). We decided to additionally publish this part of the study to provide important details on prospective trial registration of RCTs and handling options in evidence synthesis for relevant discussions in the research community. We improved the last para in the background section and the first para in the methods section:

*" **Background** (…) This article is part of a meta-epidemiological study which applies the novel and non-validated research integrity assessment (RIA) tool,[13] designed for RCTs included in evidence synthesis, to a pool of RCTs included in COVID-19 systematic reviews. The original RIA tool is available elsewhere.[13] In the present study, we focus on the assessment of the second domain of the RIA tool, i.e. prospective trial registration of RCTs. We present reporting of trial registration in the study reports of COVID-19 RCTs, provide guidance for producers of evidence synthesis on how to assess trial registration in RCTs, and discuss the feasibility of the tool for its use in evidence synthesis.*

Methods

The protocol for the meta-epidemiological study has been published, including the search for RCTs and the assessment of prospective trial registration (https://osf.io/3bzeg). We extracted and analyzed additional study data which was not prospectively planned, but designed post hoc to describe the study pool in detail. Additional analyses are indicated as such."

#5 Please add a limitations chapter in the discussion.

Response: We added the following limitation chapter to the discussion: "Our study has several limitations. First, RIA is limited to systematic reviews of more recently conducted RCTs. Second, our study is limited to a RIA of COVID-19 RCTs. Therefore, generalizability to other time periods or other medical fields is limited. Third, lack of statistical testing, considering the absence of

prospective planning, is another limitation of this work hindering strong conclusions on any reported association between study characteristics and prospective registration."

#6 "To our mind, a consensus is needed within the evidence synthesis community on whether a study pool should be restricted to prospectively registered RCTs. Currently, we argue in favor of this approach because it aligns with international standards, is essential for correctly assessing a RCT, is easy for trialists to implement, and speeds up the evidence synthesis process by excluding many small and poorly reported RCTs." –

#6.1 I would rather be cautious and I don't think that we should make generic statements like to always exclude not prospectively registered RCTs. Meta-research is needed to show if the excluded studies could change results in meta-analyses. Even if meta-research shows results that these trials might be excluded researchers should still be able to decide that for each individual systematic review as the impact might be very context-specific. I think sensitivity analyses would be a better way to deal with it rather than exclude these trials.

Response: You raise an important point regarding context-specific considerations. Indeed, for RCTs published before 2010, prospective trial registration was not a well-known international standard (2004 International Committee of Medical Journal Editors (ICMJE): Trial registration in a public trial registry before the enrolment date as a prerequisite for accepting trial publication in any ICMJE member journal; 2005 World Health Organization: Standardise the trial registration process across multiple international trial registries, with emphasis on the trial registration number (TRN); 2008 Seventh revision of the Declaration of Helsinki: Emphasis on prospective trial registration before the enrolment date; from https://www.bmj.com/content/369/bmj.m982). As such, excluding these trials may not be appropriate, and we agree that context matters. However, when it comes to trials published after 2010, adherence to international standards - particularly prospective registration - has become essential. The primary goals of prospective registration are to prevent selective reporting and to create a publicly accessible, structured database of trial information. This is crucial for ensuring the reliability of RCTs, especially those investigating Investigational Medicinal Products (IMPs).

Today, there is no justification for missing prospective registration. We, as producers of evidence synthesis, must consider this in our RI assessments. A fully reliable study must be prospectively registered. Only when such studies are no longer included in systematic reviews and guidelines due to non-compliance with international standards, a shift in perspective can be forced, affecting funding and personal reputation.

We included a sentence in the limitation section on the limitation of the RIA to systematic reviews of more recently conducted RCTs: "Our study has several limitations. First, RIA is limited to systematic reviews of more recently conducted RCTs."

#6.2 Please also discuss if a missing protocol or registration is already addressed in RoB 2 or in ROBINS-I (I think yes). Therefore, the risk of bias due to a missing registration/protocol might be already considered and could be reported as such; and also lead to downgrading of the certainty of evidence. Please discuss and revise the conclusion if appropriate.

Response: Thank you for bringing up RoB 2. As you noted, the RoB 2 tool addresses bias that arises because a reported result is selected (based on its direction, magnitude or statistical significance) from among multiple intervention effect estimates that were calculated by the trial investigators (https://sites.google.com/site/riskofbiastool/welcome/rob-2-0-tool/current-version-of-rob-2). This issue is particularly relevant when analysis intentions are unclear—such as when there is no prospectively registered protocol. In such cases, RoB 2 suggests a 'some concerns' rating for bias, as it becomes difficult to assess whether outcomes were selectively reported. We argue that non-registration or retrospective registration with undetectable selective outcome reporting could give RCTs a comparative advantage over prospectively registered RCTs where selective outcome reporting is verifiable, resulting in a "high risk of bias" rating.

Therefore, we and others argue that authenticity and integrity of RCTs should be assessed before conducting a critical appraisal of RCTs (https://www.thelancet.com/pdfs/journals/eclinm/PIIS2589-5370(24)00296-7.pdf).

The arguments under #6.1 highlighting the key goals of prospective registration together with #6.2 explaining the unreliability when assessing RCTs without pre-specified plans (e.g. registrations) for risk of selective outcome reporting underscores that there is no justification for missing prospective registration today. Therefore, RCTs (published after 2010) without prospective registration are considered problematic in terms of research integrity and should be excluded from the evidence synthesis according to RIA.

Minor comments Methods:

#1 Could you attach the RIA tool to the manuscript so readers would better understand how the tool looks like?

Response: We have referenced the RIA tool in the paper and included the information that the tool was available via this reference: "The original RIA tool is available elsewhere.[13]"

[13] Weibel S, Popp M, Reis S, et al. Identifying and managing problematic trials: A research integrity assessment tool for randomized controlled trials in evidence synthesis. Res Synth Methods 2023;14(3):357-69. doi: 10.1002/jrsm.1599

#2 "We documented the screening and selection process of systematic reviews and RCTs in a PRISMA flow diagram including reasons for exclusion at the full-text screening stage." – add Figure number.

Response: We apologize for the lack of clarity. We made it clearer: "A PRISMA flow diagram is shown in Supplemental File 3."

Results:

#3 Table 2: Could add a legend explaining what "minus days" (e.g. -3) mean and what "plus days" (e.g. 2).

Response: Thank you for that notice. We changed the item in Table 2 into "Time between registration and study start (days)[a]" and added a footnote:

[a] (…) Time was measured between submission/registration and study start. Study start was defined as time point 0. Negative days indicate 'registration/submission before study start' and positive days indicate 'registration/submission after study start'.

#4 Table 2: You could add percentages behind frequencies where appropriate.

Response: We have decided not to include percentages in the table, as the text provides context for the data based on different populations. For example, we discuss the distribution of study sizes within the assessment categories (no concern, awaiting classification, exclude) and the distribution of assessment categories within large, medium, and small studies. Therefore, the table focuses solely on summarizing the raw numbers.

#5 Table 3: The title of the table does not fit well to the categories (ICMJE, MEDLINE…). These are not journals.

Response: We have improved description of the categories in Table 3.

#6 Table 3: You could add percentages behind frequencies where appropriate.

Response: The same applies here as in the previous question #4. In the text, different populations were used (assessment categories versus journal characteristics). Thus, the table only summarizes the raw numbers.

#7 Table 3: The levels in the Norwegian Register could be explained in the legend.

Response: We added the following to the legend "The Norwegian Register's ranking system includes levels X, 0, 1, and 2. Level 1 and 2 journals are approved, with Level 2 meeting all criteria and Level 1 meeting the minimum requirements. Level 0 journals do not meet the standards, while Level X indicates uncertainty due to concerns about predatory practices."

#8 You could refer to the flow diagram at the beginning of the results chapter.

Response: It is referenced at the beginning of the results section ("A PRISMA flow diagram is shown in Supplemental File 3").

#9 The discussion would benefit from subchapters.

Response: I would suggest referring to the journal's format, as discussions in BMJ Open typically do not include subheadings. The editor may provide further instructions on this matter.

#10 Conclusions: "If prospective trial registration is required for inclusion in evidence syntheses, only six of ten COVID-19 RCTs would be eligible" – Do you have a comparison from non-Covid studies you could include in the discussion?

Response: We added a large cross-sectional analysis measuring the frequency of prospective trial registration among RCTs published in different fields of medicine in 2018: "Nevertheless, our study showed a substantial increase in prospective trial registration in COVID-19 studies compared to earlier years.[20 21] Al-Durra et al, for example, investigated about 10,000 manuscripts of RCTs published in more than 2,000 journals in 2018 and found that 42% complied with prospective trial registration.[20] In the context of RIA, evidence syntheses examining RCTs published before the COVID-19 pandemic would include even fewer prospectively registered studies, resulting in an even smaller study pool."

#11 Conclusions: "we argue in favor of this approach" – Which approach?

Response: Thank you for that notice. We tried to phrase it more clearly: "*Currently, we argue in favor of restricting the study pool to prospectively registered RCTs (in systematic reviews of more recently conducted studies) because it aligns with international standards, is easy for trialists to implement and straightforward for systematic reviewers to assess, is essential for correctly assessing bias of a RCT, and speeds up the evidence synthesis process by excluding many small and poorly reported RCTs."*

Reviewer: 2

Dr. Mohamed Rahouma, Weill Cornell Medicine, National Cancer Institute

Comments to the Author:

Pscheidl et al reported their work named "Investigation of trial registration as part of a research integrity assessment of randomized controlled trials in COVID-19 evidence syntheses" and concluded "If prospective trial registration is required for inclusion in evidence syntheses, only six out of ten COVID-19-RCTs would be eligible. Restricting eligibility to prospectively registered RCTs would include the vast majority of large and international multi-centre RCTs but exclude many smaller and non-European RCTs.". I have the following comments:

Thank you for your critical and helpful comments.

Main comment:

- Statistical Analysis: The study is described as descriptive, and no statistical hypothesis

testing was performed. While this is appropriate for the study's aims, the authors should consider whether more advanced statistical methods (e.g., regression analysis) could provide additional insights into factors associated with prospective registration.

Response: Thank you for your thoughtful comment. We agree that further statistical hypothesis testing could potentially provide additional insights. However, as noted in the methods section, "we did not perform any statistical hypothesis testing, as this part of the study was not prospectively planned but designed post hoc to disseminate relevant findings." We believe that conducting post hoc statistical tests that were not pre-planned is problematic, as such analyses are highly susceptible to selective outcome reporting and p-hacking. As strong advocates for prospective registration and adherence to pre-specified analysis plans, we strive to follow this approach in our own work. We have mentioned the lack of statistical testing, considering the absence of prospective planning, as a limitation in the discussion.

- Data Availability and Transparency: The authors mention that they contacted study authors for missing or inconsistent registration details. However, the response rate was only 25%. The manuscript should discuss the potential impact of non-response on the study's findings and whether alternative methods could be used to obtain missing data.

Response: Thank you for your thoughtful comment.

We identified 17 RCTs which did not report a trial registration number (TRN). We identified 4 TRN via active search (3 of 4 retrospective), and 13 authors of RCTs were requested (one retrospective, and one not registered) of which 11 did not respond. We may conclude that the majority of RCTs without reported TRN were not or were retrospectively registered.

We identified 31 RCTs with missing or inconsistent information. Author requests helped to classify seven studies as prospective registrations and two as retrospective. We may conclude that we may have missed at least some prospectively registered RCTs due to insufficient reporting or lack of updating study information regarding registration details and study dates.

We contacted all authors of inconclusive RCTs and conducted additional searches for the registrations of all RCTs that either lacked a reported TRN or had retrospective registrations. We believe that responsiveness in correspondence is a key indicator of trustworthiness, while a lack of response undermines it. Accountability and transparency are essential for research integrity. RCTs that fail to transparently report important trial registration details or refuse to share this information upon request raise concerns about their research integrity.

We added the following para to the discussion section: "We contacted the authors of 44 RCTs that either lacked a reported trial registration number or had inconsistent or missing information regarding registration or study dates. However, the response rate was only 25%. Out of the 11

RCTs that did respond, seven could be classified as prospectively registered. This suggests a risk that a significant number of inconclusive RCTs are prospectively registered but may have been incorrectly excluded in the RIA. We believe that responsiveness in correspondence is a key indicator of trustworthiness, while a lack of response undermines it. Accountability and transparency are crucial for research integrity. RCTs that fail to transparently report essential trial registration details or refuse to share this information upon request raise concerns about their research integrity."

Other comments:

- Clarity and Justification of the RIA Tool: The authors use the Research Integrity Assessment (RIA) tool, which is described as novel and non-validated. While the tool appears comprehensive, the manuscript would benefit from a more detailed discussion of its development, validation process, and how it compares to existing tools for assessing research integrity. Additionally, the authors should justify why this tool was chosen over other established methods for assessing trial registration.

Response: Thank you for your comment. We believe that we were not precise enough stating the purpose of our study as Reviewer #1 mentioned a similar comment (#4).

The present study is part of a larger meta-epidemiological study investigating the impact of the RIA tool (currently unpublished). We have developed the RIA tool and published it in 2022. We referenced the protocol to the meta-epidemiological study (https://osf.io/3bzeg). We decided to additionally publish this part of the study to provide important details on prospective trial registration of RCTs and handling options in evidence synthesis for relevant discussions in the research community. We improved the last para in the background section and the first para in the methods section to inform the reader about the aim and purpose of the present study:

" Background (…) This article is part of a meta-epidemiological study which applies the novel and non-validated research integrity assessment (RIA) tool,[13] designed for RCTs included in evidence synthesis, to a pool of RCTs included in COVID-19 systematic reviews. The original RIA tool is available elsewhere.[13] In the present study, we focus on the assessment of the second domain of the RIA tool, i.e. prospective trial registration of RCTs. We present reporting of trial registration in the study reports of COVID-19 RCTs, provide guidance for producers of evidence synthesis on how to assess trial registration in RCTs, and discuss the feasibility of the tool for its use in evidence synthesis.

Methods

The protocol for the meta-epidemiological study has been published, including the search for RCTs and the assessment of prospective trial registration (https://osf.io/3bzeg). We extracted

and analyzed additional study data which was not prospectively planned, but designed post hoc to describe the study pool in detail. Additional analyses are indicated as such."

We have referenced the RIA tool in the paper and included the information that the tool was available via this reference: "The original RIA tool is available elsewhere.[13]"

[13] Weibel S, Popp M, Reis S, et al. Identifying and managing problematic trials: A research integrity assessment tool for randomized controlled trials in evidence synthesis. Res Synth Methods 2023;14(3):357-69. doi: 10.1002/jrsm.1599

We also included a para in the discussion comparing RIA with two other tools:

"We face the challenge of how to handle studies without prospective registration in research integrity assessments conducted within evidence syntheses. In RIA, all RCTs without prospective registration are excluded, regardless of other aspects such as ethics or data trustworthiness. We have chosen a hierarchical approach to work more efficiently. This approach was based on the assumption that restricting to prospective RCTs would not result in the loss of large, well-conducted trials. In contrast, TRACT, another trustworthiness checklists, assesses RCTs without prospective registration (and published after 2010) as 'major concern' triggering a more thorough investigation, including assessment of original individual participant data.[3] A third Trustworthiness Screening Tool (TST) developed by the Cochrane Pregnancy and Childbirth Group places RCTs without prospective registration (and published after 2010) in the "awaiting classification" category, meaning they do not contribute to evidence synthesis findings.[4] The key question for the research community is whether the study pool should be restricted to prospectively registered RCTs or whether prospective registration should be viewed as part of a broader, more holistic approach in a research integrity assessment, encompassing ethics and governance, to prevent the exclusion of relevant RCTs."

- Generalizability of Findings: The study focuses exclusively on COVID-19 RCTs, which may limit the generalizability of the findings to other therapeutic areas. The authors should discuss whether the observed trends in trial registration are likely to be similar in non-COVID-19 trials or if the urgency of the pandemic may have influenced registration practices differently.

Response: We extended the discussion on time trends of prospective registration: "Nevertheless, our study showed a substantial increase in prospective trial registration in COVID-19 studies compared to earlier years.[20 21] Al-Durra et al, for example, investigated about 10,000 manuscripts of RCTs published in more than 2,000 journals in 2018 and found that 42% complied with prospective trial registration.[20] In the context of RIA, evidence syntheses examining RCTs published before the COVID-19 pandemic would include even fewer prospectively registered studies, resulting in an even smaller study pool."

Additionally, we added the restriction to COVID-19 RCTs as a limitation of our study to the discussion: "Second, our study is limited to a RIA of COVID-19 RCTs. Therefore, generalizability to other time periods or other medical fields is limited."

- Handling of Retrospective Registrations: The authors classify RCTs as "retrospectively registered" if registration occurs after the study start date. However, they note that some registries (e.g., in the US and UK) allow registration within 30 days of study initiation. The manuscript would benefit from a more nuanced discussion of how different regulatory environments might impact the classification of retrospective registration and whether a uniform standard (e.g., WHO/ICMJE) should be applied globally.

Response: Thank you for your valuable comment. While we appreciate the suggestion for a more nuanced discussion, we believe our current discussion is already comprehensive, and further elaboration might extend beyond the scope of this paper. We have highlighted that "16 RCTs were registered within 30 days of study initiation, in line with US and former UK regulations. [22 23] *In contrast, we used the WHO and ICMJE definition of prospective registration which means registration before enrollment of the first participant.*[7 8] *In this respect, international harmonization of clinical trials regulation would be helpful for classification." We hope that this information helps readers understand the potential impact and that this information may trigger international standardization.*

- Impact of Excluding Non-Prospectively Registered RCTs: The authors suggest that excluding non-prospectively registered RCTs would exclude many smaller and non-European studies. While this is an important finding, the manuscript would benefit from a deeper exploration of the potential consequences of such exclusions. For example, how might this impact the diversity of evidence in systematic reviews, and could it lead to biases in the evidence base?

Response: This is an excellent comment, though it's a difficult question to answer. On one hand, excluding non-prospectively registered RCTs could reduce the diversity of the evidence base, particularly regarding ethnicities investigated in smaller RCTs from non-European countries, as these are often not prospectively registered. On the other hand, excluding such studies enhances the trustworthiness of the evidence base. We believe the solution lies in encouraging all trialists to prospectively register their RCTs. Almost all large international multi- center RCTs were prospectively registered! Allowing exceptions would not improve the situation in the long run. Currently, there is no justification for missing prospective registration. Prospective registration can be done with minimal financial and personnel resources from anywhere in the world in national or international registries. We as producers of evidence synthesis must consider this when conducting research integrity assessments. A fully reliable study must be prospectively registered. By excluding studies that do not comply with international standards,

we can drive a shift in perspective, which could impact funding and personal reputation. We advocate to include only prospectively registered RCTs (if published after 2010) in systematic reviews, as this aligns with international standards, is easy for trialists to implement, and accelerates the evidence synthesis process by excluding poorly reported studies.

We have added a sentence to the discussion emphasizing the need to explore the impact of restricting to prospectively registered RCTs on the diversity of the evidence base: "However, future studies should examine the consequences of such restrictions on diversity of the evidence base."

- Journal Policies and Compliance: The authors assess whether journals follow ICMJE recommendations for prospective trial registration but find that 30-40% of RCTs in ICMJE-compliant journals are retrospectively registered. This raises questions about the enforcement of journal policies. The authors should discuss potential reasons for this discrepancy and suggest ways journals could improve compliance with registration requirements.

Response: Thank you for this valuable comment. We agree that the points you raised are important and deserve further discussion. However, space in our discussion is limited, and a deeper exploration of this issue would extend the scope of our work. The reasons behind the discrepancy between ICMJE recommendations and the publication of many retrospectively registered RCTs in journals following the ICMJE recommendations are indeed complex and warrant further attention. Our intention here is to raise awareness of this issue, while the potential solutions should be explored in other contexts. We have chosen to focus on the key aspects to maintain the focus of the study. We hope that future research will further investigate these questions.

- Ethical Considerations: The manuscript briefly mentions ethics approval but does not explore the ethical implications of non-prospectively registered trials in depth. Given that prospective registration is an ethical requirement in many jurisdictions, the authors should discuss the ethical dimensions of their findings, particularly in the context of patient safety and transparency.

Response: Thank you for this important comment. We agree that the ethical implications of non-prospectively registered trials are a significant issue, especially in relation to patient safety and transparency. We have indicated this in the background section. However, due to space limitations, we are unable to explore this topic in depth within the current manuscript - though we have published another manuscript on the topic (Investigation of ethics approval as part of a research integrity assessment of randomised controlled trials in COVID-19 evidence syntheses: a meta-epidemiological study | BMJ Open). Our primary aim in the context of this manuscript

was to highlight the issue and raise awareness, while a more detailed discussion of the ethical dimensions would be better suited for future work. We hope that this will encourage further exploration of these critical concerns in the broader context of clinical research.

- Discussion of "Retroactively Prospective" Trials: The authors identify two RCTs that changed their study start dates to appear prospectively registered. This is a concerning finding that warrants further discussion. The authors should explore the potential motivations for such changes and suggest ways to prevent this practice in the future.

Response: Thank you for your valuable comment. We agree that the identification of 'retroactively prospective' trials is a concerning finding that warrants further discussion. The term itself is relatively new, and we referenced a recently published study that introduced this terminology. To our knowledge, we are among the first to identify "retroactively prospective" RCTs included in evidence synthesis.

The recently published study also discussed reasons for 'retroactively prospective' registrations and we cited this in our discussion: "A recent study measured the rate of 'retroactively prospective' trials in ClinicalTrials.gov in 2015,[24] and identified 2% of all clinical trials in a sample of 11,908 trials. While these changes to the start date could be mistakes or legitimate edits based on the most up-to-date information, they could also indicate a retrospectively registered trial that has been made to appear as a prospectively registered trial, which represents scientific flaw and would lead to biases unapparent to producers of evidence syntheses.[24] For RI assessments in evidence synthesis, we need a consensus on handling 'retroactively prospective' RCTs in evidence synthesis."

- Recommendations for Evidence Synthesis Producers: The authors conclude that a consensus is needed on whether to restrict evidence syntheses to prospectively registered RCTs. While this is a valid point, the manuscript would benefit from more specific recommendations for systematic reviewers. For example, should reviewers always exclude non-prospectively registered trials, or are there circumstances where they might be included with appropriate caveats?

Response: Thank you for your comment. It is important to note that we are still far from providing definitive recommendations for systematic reviewers, as there are many unresolved questions in this area. In our manuscript, we aimed to highlight these uncertainties and open the floor for further discussion within the research community. Some of the key questions we believe need to be addressed are (included and discussed in the manuscript):

1. *What constitutes <u>prospective</u> registration, given the lack of international harmonization in clinical trial regulations (e.g. before participant enrolment, within 30 days or 6 weeks, before outcome measurement, or before study completion)?*

2. *How should multiple registrations or unclear primary sites in multi-center RCTs be handled?*
3. *How should 'retroactively prospective' RCTs be treated in evidence synthesis?*
4. *What is the impact of restricting evidence syntheses to prospectively registered RCTs on the diversity of the evidence base?*
5. *How should RCTs initiated after the WHO/ICMJE Declaration of mandatory registration in 2005 (ICMJE | About ICMJE | Clinical Trials Registration), or published before 2010, be handled?*
6. *A key question for the research community is whether the study pool should be restricted to prospectively registered RCTs or whether prospective registration should be viewed as part of a broader, more holistic approach in a research integrity assessment, encompassing ethics and governance, to prevent the exclusion of relevant RCTs?*

We hope that these open questions will encourage further debate and contribute to developing clearer guidelines for systematic reviewers in the future.

- Limitations: The authors acknowledge several limitations, including the focus on trial registration and the reliance on ClinicalTrials.gov for submission dates. However, they should also discuss the potential impact of these limitations on the study's conclusions and suggest ways future research could address these issues.

Response: We respectfully disagree with your critique. The reliance on submission dates from ClinicalTrials.gov for determining prospective registration is not a limitation of our study. In fact, it is a clear strength. We demonstrate the importance of registries providing submission dates for accurate assessments of prospective registration, and we encourage other registries to follow ClinicalTrials.gov's example. The focus on trial registration in the submitted manuscript was chosen to share detailed insights from our meta-epidemiological study on RIA, particularly in the area of prospective trial registration, with the research community. However, readers should recognize that research integrity extends far beyond just the prospective registration of a trial.

<u>Reviewer: 3</u>

Prof. Andrew Jull, The University of Auckland, The University of Auckland

Comments to the Author:

Thank you for the opportunity to read this interesting paper. The purpose is to assess whether trial registration is an element that should be considered in assessing the quality of a RCT when including the same in systematic review. I have no major concerns about this paper, but raise a few minor issues that need resolution.

Thank you for your critical and helpful comments. We would like to kindly point out that we are not assessing the quality of a study, but rather evaluating the integrity of the study.

1. Abstract and elsewhere: It is not clear to me whether the conclusions are limited to trials associated with COVID interventions and are more generalisable. I suspect the authors are aiming for the latter, but if so, will need to generalise the conclusions (especially in the abstract), which appear limited to inferences that might be drawn from COVID trials alone. I do think that that if the authors are aiming at more generalisable conclusions as indicated in the conclusions on page 20, then that attempt is precipitate, given the only data then have tested their hypotheses upon are trials involving COVID and other groupings of trials needed to be evaluated before the evidence synthesis community could be asked for a consensus on using prospective trial registration as an inclusion criteria. However, it certainly makes sense to consider prospective trial registration as a marker for sensitivity analyses, but not a for an in/out decision at this point in time.

Response: Thank you for your thoughtful comment. Our study focuses specifically on COVID-19 RCTs included in evidence syntheses. At this stage, we are cautious about extending the findings of this study to all RCTs, particularly those published before 2010. Prior to 2010, prospective trial registration was not widely known as an international standard (e.g., ICMJE 2004, WHO 2005, or the 2008 Declaration of Helsinki). Therefore, excluding these trials may not be appropriate, and we agree that context is crucial. We added this as a limitation of our study to the discussion.

However, for trials published after 2010, adherence to international standards, especially prospective registration, has become essential. The main goals of prospective registration are to prevent selective reporting and ensure the availability of structured, publicly accessible trial information. This is especially important for ensuring the reliability of RCTs, particularly those involving Investigational Medicinal Products (IMPs).

Our study utilizes the RIA tool, which was originally developed to assess the research integrity of ivermectin RCTs during the pandemic (Weibel et al., 2022). Prospective registration is just one integrity domain of overall six domains. The RIA tool initially suggested excluding COVID- 19 RCTs without prospective registration (domain 2). This study extends domain 2 of this tool (without modification!) to a broader range of COVID-19 RCTs, allowing us to critically assess and test the tool's domain 2 (prospective registration). We hope our findings, open questions, and discussions will serve as a foundation for further conversations in the research community, as we highlighted in our manuscript: e.g. "The key question for the research community is whether the study pool should be restricted to prospectively registered RCTs, or whether prospective registration should be part of a more holistic approach in a research integrity assessment, encompassing ethics and governance, to prevent the exclusion of relevant RCTs."

Moving forward, the RIA tool will be adapted in future based on new findings and ongoing discussions within the community.

2. Page 4: GRADE is not a critical appraisal tool, but rather is a means for evaluating the level of certainty associated with an evidence statement guiding the type of language that should be used in the evidence statement. Also why is inter in brackets for international on line 26?

Response: Thank you for your thoughtful comment. Critical appraisal in the context of evidence synthesis refers to the process of evaluating amongst others, the quality, heterogeneity, relevance, and credibility of studies (and their results) included in a synthesis (e.g., systematic review or meta-analysis). It involves assessing study design, methodology, bias risks, and the applicability of results to the research question. This ensures that conclusions drawn from the synthesis are based on reliable and high-quality evidence. As you mentioned, GRADE is used to evaluate the certainty of evidence, but it's not a critical appraisal tool. Rather, it's an approach that involves a critical appraisal of study results, considering factors like risk of bias, indirectness, imprecision, inconsistency, and publication bias. You're right - GRADE is an approach, not a tool.

We corrected the sentence "Critical appraisal tools, like the Cochrane Risk of Bias tool 2 (RoB 2), and approaches such as the Grading of Recommendations, Assessment, Development and Evaluation (GRADE), evaluate the internal and external validity of study results.[1] [2] However, they do not necessarily address aspects of research integrity."

Regarding your second point. We removed the brackets and revised the sentence into "Most researchers associate research integrity with the use of honest and verifiable methods in proposing, performing, and evaluating research, but research integrity also comprises adhering to national, international and commonly accepted guidelines, regulations, norms or standards."

3. Please provide more clarity in the last sentence in paragraph 3 on page 6. I did not understand the point the authors were trying to make.

Response: We hope this clarification addresses the sentence in question: "Multiple primary study reports of a study (e.g. journal publication and preprint) were not pooled for our assessment but were separately assessed as included in the original systematic review." We apologize for any confusion caused by our wording. What we meant to convey is that we did not combine different reports of the same study (such as journal publications, preprints, and trial registration records) identified in the various systematic reviews. Instead, each report was assessed separately, as it was included in the original systematic review. This means that, in some cases, a study may have been assessed more than once in our meta-epidemiological study - once as a preprint and again as a journal publication, for example.

We revised the sentence into "We did not combine different reports of the same study (such as

journal publications, preprints, and trial registration records) identified in the various systematic reviews. Instead, each report was assessed separately, as it was included in the original systematic review."

In our study, there were two RCTs reported as preprint and journal publication(s). Gupta- 2021a, COMET-ICE and Gupta-2021b, COMET-ICE; Weinreich-2021a, (phase 1-2), Weinreich-2021b, (phase 1-2), and Weinreich-2021c, (phase 3). However, all reports differed in the number of analyzed participants: i.e. 583, 1057, 275, 799, 5607 (e.g. interim analysis and completed study analysis). For details see https://doi.org/10.17605/OSF.IO/87UT4

4. Line 24 page 8 and through out: please spell out in full prospectively instead of "pro- or retrospectively...".
5. Lines 10-12, page 9: I could not understand the sentence - please provide more clarity.

Response: We apologize for the lack of clarity. We have revised the paragraph to the following for better understanding: "We conducted a search from August 28th to August 30th, 2023, to check whether the journal was listed on the ICMJE website. The listed date on the website was considered the start date when the ICMJE guidelines were included in the journal's editorial policies. If the start date or the journal was not listed on the ICMJE website, we gathered the information either by checking the journal's homepage or by contacting the journal's editorial team via E-mail."

6. Page 13, line 47: The ISRCTN is the UK's trial register and is owned by the the non-profit company ISRCTN and operated by Springer Nature on their behalf.

Response: Thank you for spotting this mistake. We mistakenly confused the UK's trial register ISRCTN with the WHO International Clinical Trials Registry Platform (ICTRP) in this sentence.

7. Page 14, line 30: please provide detail of prospective registration for the >100<200 group of trials.

Response: Thank you for this notice. All information is provided in Table 2. We added the reference for Table 2 to the sentence.

8. Table 2: For setting and location, all locations are multinational continents, except for Australia. Was it your intention to only include Australia or did you mean Australasia (Australia and New Zealand) or Oceania (Australia, NZ, Pacific nations). Further, given there were 0 trials in Australia, why include it at all?

Response: Thank you for your valuable comment. We were not precise in this instance. You are correct that there were 0 RCTs from this continent, and we have decided to remove it from the table.

9. Page 16, line 29: revise to read "three either not or retrospectively registered..."

Response: We have made the correction as suggested.

10. Discussion first paragraph and conclusion: please revise page 18 line 7 and page page 20 line 47. It is not the case that every 10th trial did not report registration details or study date insufficient on every 7th trial), which would mean that trials 10, 20, 30, 40, etc (or 7, 14, 21, 28, etc) were affected. Instead you mean one in 10 trials (or one in seven trials) were so affected.

Response: Thank you. We have revised the first and the last paragraph of the discussion to be correct and precise: "In our assessment including 188 COVID-19 RCTs nine out of ten reported at least one trial registration number, and one in ten RCTs did not report any registration details.", "If prospective trial registration is required for inclusion in evidence syntheses, only six of ten COVID-19 RCTs would be eligible. Reporting of registration details and study dates was insufficient in 15% of RCTs, and 27% of RCTs were not or retrospectively registered."

11. Page 18, line 30: Do you mean regulation or registration?

Response: We mean regulation. The sentence is in line with the para discussing differences in national regulations regarding prospective registration: "Definitions of prospective registration vary internationally, hampering classification for evidence synthesis producers. Among the 39 retrospectively registered RCTs, 16 were registered within 30 days after the study start, <u>aligning with US and UK regulations</u>.[22 23] In contrast, we used the WHO and ICMJE definition of prospective registration which means registration before enrollment of the first participant.[7 8] In this respect, international harmonization of <u>clinical trials regulation</u> would be helpful for classification."

12. Page 19, line 41: is it appropriate to use correlate when no such association has been established with significance testing? Also line 44 - include "is" after placed.

Response: You are right. We revised the sentence into "Only publication in level 2 journals of the Norwegian Register appears to be associated with prospective registration.".

We added the "is" as suggested.

13. Revise page 19, lines 58-60 to read "The key question is not whether prospective registration should be an isolated exclusion criterion, but whether it should be considered..."

Response: The sentence you refer to is now revised according to comments from the other reviewers.

The para now reads: "We face the challenge of how to handle studies without prospective registration in research integrity assessments conducted within evidence syntheses. In RIA, all RCTs without prospective registration have been excluded, regardless of other aspects such as ethics or data trustworthiness. We have chosen a hierarchical approach to work more efficiently.

This approach was based on the assumption that restricting to prospective RCTs would not result in the loss of large, well-conducted trials. In contrast, TRACT, another trustworthiness checklist assesses RCTs without prospective registration (and published after 2010) as 'major concern' triggering a more thorough investigation, including assessment of original individual participant data.[3] A third Trustworthiness Screening Tool (TST) developed by the Cochrane Pregnancy and Childbirth Group places RCTs without prospective registration (and published after 2010) in the "awaiting classification" category, meaning they do not contribute to evidence synthesis findings.[4] The key question for the research community is whether the study pool should be restricted to prospectively registered RCTs or whether prospective registration should be viewed as part of a broader, more holistic approach in a research integrity assessment, encompassing ethics and governance, to prevent the exclusion of relevant RCTs."

14. Page 20, lines 17-21: You raise a reasonable point, but do not consider [a] whether an retrospectively registered trial (or indeed) an unregistered trial with a published protocol has such a theoretical advantage; [b] whether trials published before either registers were available (1999-2000 for ISRCTN and clinical trials.gov) or before registration was announced as mandatory (Sept 2004) and made mandatory for participating journals that made up the ICMJE at the time (Sept 2005); and [c] the Cochrane RoB v2 (RoB2) tool actually asks about whether data was analysed in accord with a pre-specified analysis plan (or a protocol at a pinch), which are rarely available on trials registration pages and thus simply comparing lists of outcomes in a publication and a trials registry is insufficient to gain a pass in RoB2, so most trials will not gain a theoretic advantage according to the RoB2 algorithm (https://drive.google.com/file/d/1Q4Fk3HCuBRwIDWTGZa5oH11OdR4Gbhdo/view) and will be scored "some concerns" on the selected outcome domain leading to an overall assessment of some concerns even if all other domains are low risk. So I believe that the case you make is an overstated risk.

Response: Thank you for your thoughtful comment.

You are right. The RoB 2 tool actually asks about whether data was analysed in accordance with a pre-specified analysis plan (or a protocol at a pinch).

To your points [a and c]: Very good point! Thank you! Following your thoughts, we checked all our non- or retrospectively registered RCTs (n = 51) for prospective study protocols referenced within the study report (journal publication or preprint). 38 out of 51 did not reference a study protocol, while 13 referenced a study protocol. We checked the date of the referenced study protocols and checked whether the study protocols were dated before enrolment of the first participant. Four RCTs with study protocols did not report the date of the study protocol in the supplement (Bar 2021, Salvarani 2021, Sekine 2021, Ulrich 2020), in one RCT the appendix with the study protocol was not available (Kim 2021) one RCT referenced to a supplement which

does not include a study protocol (Vallejos 2021), and one RCT published a study protocol after study start and no date of submission or other protocol date is available (Devos 2022). One RCT - published in the BMJ - referenced a study protocol which was dated two months after study start (Veiga 2021). However, five RCTs – published in NEJM or JAMA journals – referenced study protocols in journal supplements which date before the start of the study (Libster 2021, Menichetti 2021, Murai 2021, Ortigoza 2022, Tang 2020). All (four) except Menichetti 2021 registered their study protocols within 30 days of study start. Menichetti 2021 registered the study after study completion. In theory, five out of 51 non- or retrospectively registered RCTs have referenced a study protocol within the journal publication which should be used for RoB 2 assessment of domain 5 (selective outcome reporting). However, 46 of 51 non- or retrospectively registered RCTs have not referenced a prospective study protocol. All of the 46 were rated as 'some concerns' in the RoB 2 domain 5. In contrast, 109 prospectively registered RCTs identified in our study could be assessed for selective outcome reporting with RoB 2, and theoretically, there is a chance to detect selective outcome reporting resulting in 'high risk of bias' assessment. Therefore, we do not assume that it is actually an "overstated risk" that non- or retrospectively registered RCTs may have a comparative advantage over prospectively registered RCTs in RoB 2 domain 5 assessment.

To your point [b]: The RIA tool is designed for recently conducted RCTs, due to trial registration being required. Another tool, INSPECT-SR is being developed which includes trial registration, and ethics approval, as among a number of items which taken all together can be used to assess the trustworthiness of a trial. These research integrity tools are to be used as part of the study selection process.

We revised the para in the discussion with adding explanations and careful wording: "The fact is that, studies without a prespecified analysis plan (or a protocol at a pinch), which most non- or retrospectively registered studies fall into, cannot be reliably assessed for risk of bias with the Cochrane RoB 2 tool,[2] especially for the domain of selective outcome reporting, giving them theoretically a comparative advantage over prospectively registered studies. Only prospectively registered studies allow for the identification of selective outcome reporting resulting in a 'high risk of bias' assessment, meaning that non- or retrospectively registered studies can never be rated as high risk of bias in this domain."

Reviewer: 1

If you have selected 'Yes' above, please provide details of any competing interests.: Not applicable.

Reviewer: 2

If you have selected 'Yes' above, please provide details of any competing interests.: Not applicable

Reviewer: 3

If you have selected 'Yes' above, please provide details of any competing interests.: Not applicable

VERSION 2 - REVIEW

**Reviewer**	**3**
**Name**	**Jull, Andrew**
**Affiliation**	**The University of Auckland, School of Nursing**
**Date**	**15-Apr-2025**
**COI**	

The authors have addressed my concerns.
